# *PMS2* germline mutation c.1577delA (p.Asp526Alafs^∗^69)-induced Lynch syndrome-associated endometrial cancer: A case report

**DOI:** 10.1097/MD.0000000000018279

**Published:** 2019-12-20

**Authors:** Man-Hua Cui, Xi-Wen Zhang, Tong Yu, Dong-Wei Huang, Yan Jia

**Affiliations:** a Department of Gynecology; b Department of Orthopedics; c Pathology department, The Second Hospital of Jilin University, Changchun, Jilin Province, China.

**Keywords:** EC, Lynch syndrome, nonsense mutation, PMS2 gene

## Abstract

**Rationale:**

Lynch syndrome (LS) is an autosomal dominant cancer predisposition condition caused by germline heterozygous mutations in mismatch repair (MMR) genes. However, as one of the MMR genes, PMS2 mutation-induced LS-associated endometrial cancer (LSAEC) was rarely reported.

**Patient concerns:**

A 26-year-old female patient suffered from prolonged menstrual period and increased menstrual flow for 2 months.

**Diagnoses:**

The patient was diagnosed with cervix CIN III, endometrial cancer (EC), anemia, and LS.

**Interventions:**

Total hysterectomy, bilateral salpingectomy, pelvic lymphadenectomy were performed for treating EC, while ovariectomy was refused by the patient. The patient underwent postoperative chemotherapy with paclitaxel combined with carboplatin for 6 courses of treatment. Laparoscopic partial enterectomy was applied for treating colon cancer 5 years later after the surgery treatment for EC. Besides, Sanger sequencing and high-throughput genome sequencing were employed to detect the genetic status of the family that included two generations with four members. Immunohistochemistry (IHC) staining was used to identify the function of PMS2 mutation.

**Outcomes:**

The 26-year-old Chinese patient suffered from LSAEC and recovered well after surgery. A PMS2 germline heterozygous mutation (c.1577delA) was confirmed by gene sequencing 5 years later. In addition, PMS2 mutation was verified by IHC. The patient was followed up for 7 years.

**Lessons:**

Carrying PMS2 germline mutation (c.1577delA) confers an extremely high susceptibility of suffering from LS-associated cancers. Thus, close clinical monitoring and prophylactic surgery are highly recommended to reduce the morbidity and mortality of LS-associated cancers.

## 1 Introduction

Lynch syndrome (LS), also known as hereditary nonpolyposis colorectal cancer syndrome, is an autosomal dominant genetic disease caused by mutations in DNA mismatch repair (MMR) genes.^[1,2]^ The role of MMR includes three aspects: maintaining the fidelity of DNA during the process of replication, reducing the occurrence of microdeletion and microinsertion caused by the decline in DNA polymerase or missense mutation during the process of replication and folding, and maintaining the stability of DNA.^[3,4]^ MMR genes mainly include MLH1, MSH2, MSH6, and PMS2. Previous studies showed that MLH1 and MSH2 mutations account for 90% of LS, MSH6 mutations for 10%, and PMS2 mutations for only 5% to 6%.^[5,6]^ These gene mutations will increase the incidence of colorectal cancer, EC, epithelial ovarian cancer, breast cancer, bladder cancer, renal cancer and gastric cancer among family members.

LS patients accounted for about 2% to 3% of all EC patients. EC is the most common parenteral neoplasm among LS patients, and the first clinical symptom in about 50% of female LS patients.^[7,8]^ Therefore, LSAEC has gradually become one focus in the medical field. The offspring of LS patients will have a 50% chance of inheritance, and most of the mutations in patients are inherited from their parents. However, there is an incomplete penetration rate in LS and reasonable risk management can reduce the risk of related cancers.

Universal screening for LS among EC patients has been recommended by numerous experts and specialist societies.^[9]^ There is evidence that EC is often a sentinel cancer for women with LS.^[10]^ Here in this paper, the diagnosis and treatment measures of a rare LS case caused by c.1577delA (p.Asp526Alafs^∗^69) mutation of PMS2 gene are reported.

## 2 Ethical approval

Patient has provided informed consent for publication of the case. This report was approved by the ethics committee of the Second Hospital of Jilin University, Changchun, China.

## 3 Case report

### 3.1 Patient

A 26-year-old Chinese female patient was diagnosed with CIN III, who underwent cervix conization.

**Figure 1:**
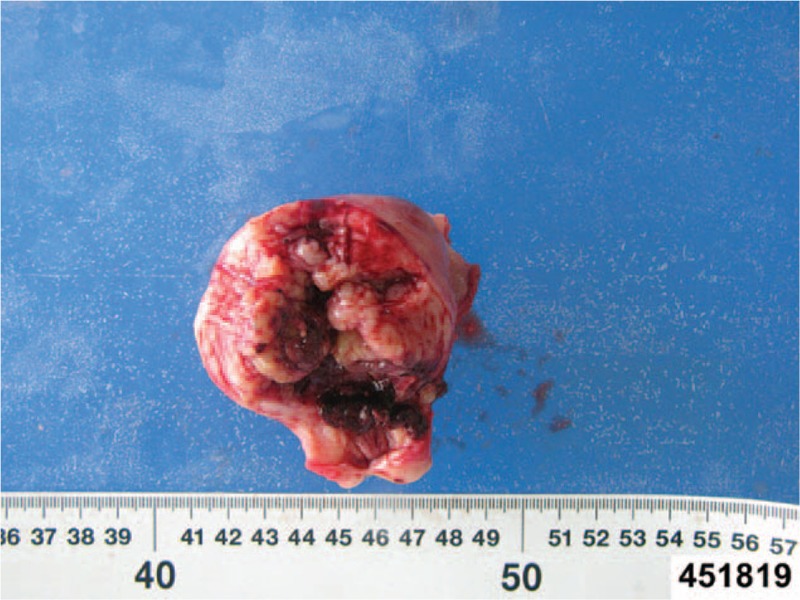
The specimen of endometrial cancer.

**Figure 2:**
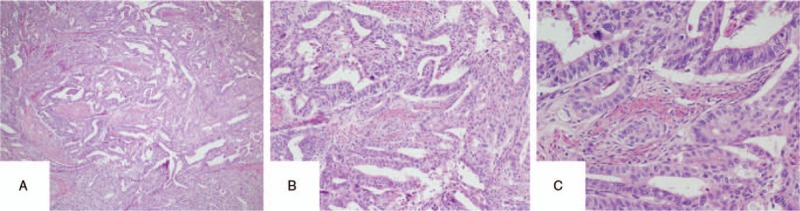
The HE staining of endometrium. (A) the microscope magnifying × 40, (B) the microscope magnifying × 100, (C) the microscope magnifying × 200.

**Figure 3:**
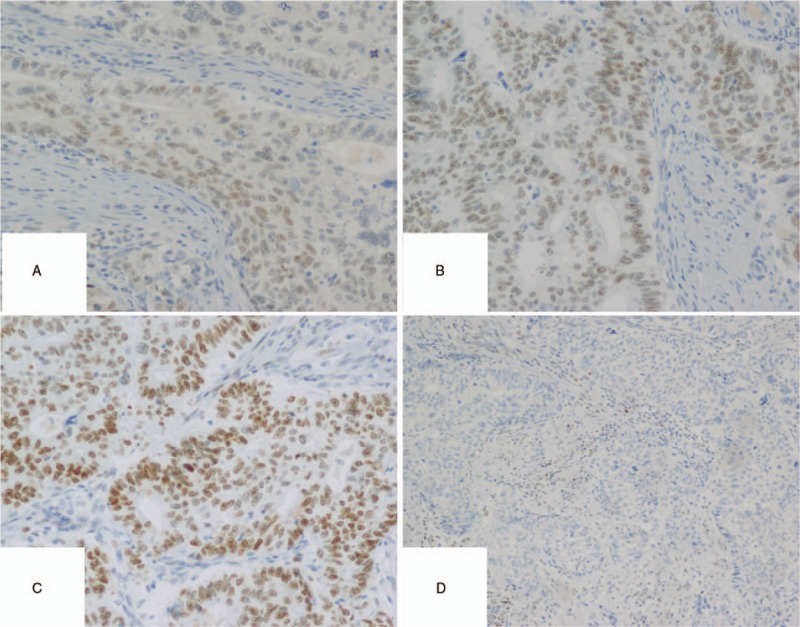
The IHC of endometrium. (A) MLH1 results showed positive nuclei, (B) MLH2 results explained positive nuclei, (C) MSH6 results demonstrated positive nuclei, (D) PMS2 results showed negative nuclei.

One year after the operation, the patient was diagnosed with EC. After ineffective conservative treatment with high-efficiency progesterone, total abdominal hysterectomy, bilateral salpingectomy, and pelvic lymphadenectomy were performed. The patient refused to have her ovaries removed, and was treated with paclitaxel (Nanjing Green Leaf Pharmaceutical Co., Ltd., China) combined with carboplatin injection (Qilu Pharmaceutical Co., Ltd., China) chemotherapy for 6 courses of treatment. Postoperative pathological (Figs. 1–3) findings were as follows: It conforms to the case of endometrioid adenocarcinoma, infiltrates the superficial layer of muscular wall, and has dense interstitial cells.

**Figure 4:**
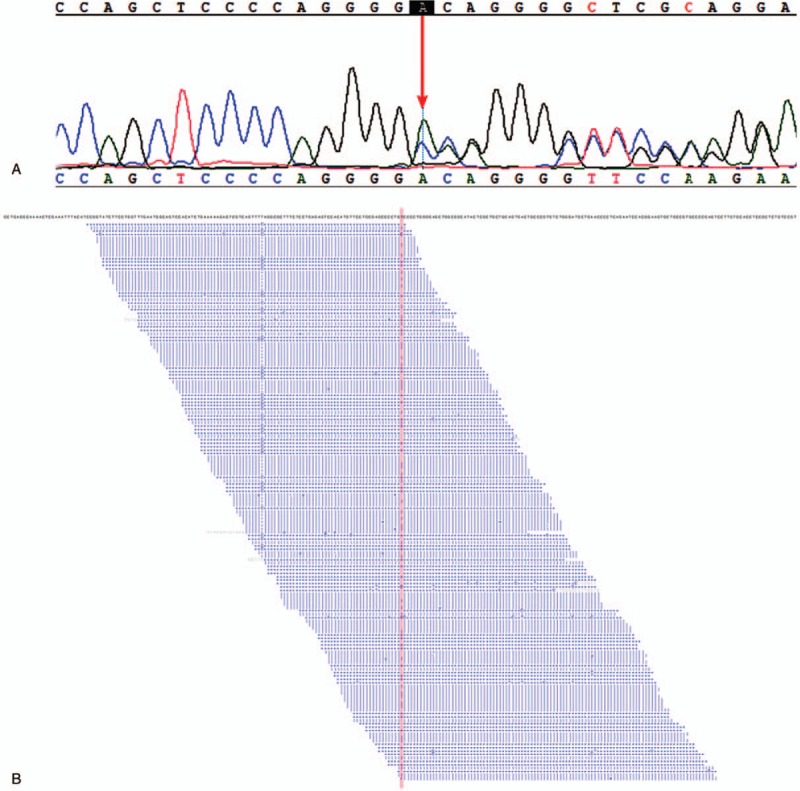
(A) The results of gene detection (Sanger Deoxygenation Chain Termination Method). A frame shift mutation c.1577 delA (p.Asp526 Alafs^∗^69) was detected in PMS2 gene of the subjects (red arrow). (B) The outcomes of high-throughput genome sequencing, and the red vertical line represent gene mutation sites.

**Figure 5:**
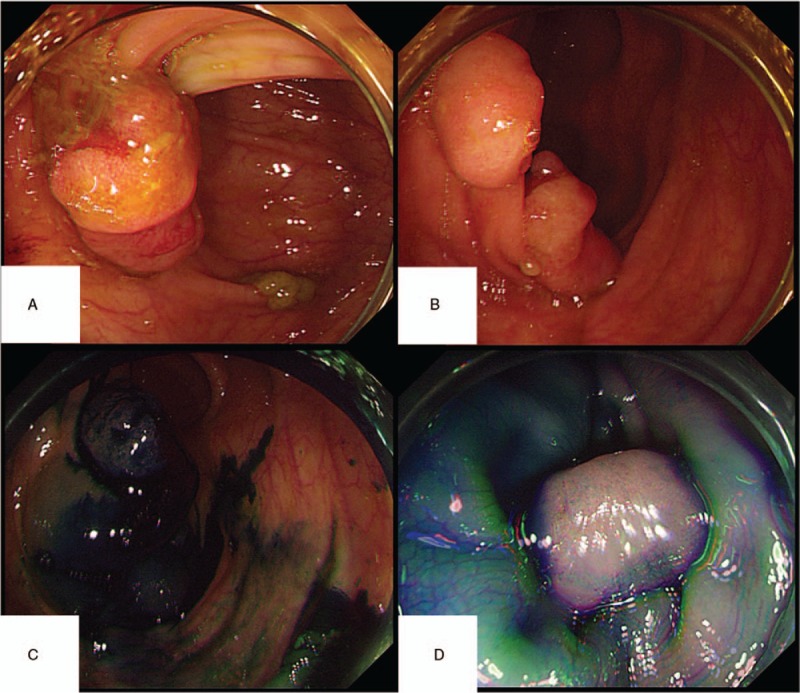
Colonoscopy pictures. There are 2 polyps in the transverse colon, about (A) 1.0 cm in size and (B) 2.5 cm in size, respectively. Submucosal injection of methylene blue at the base of lesion (C-D).

**Figure 6:**
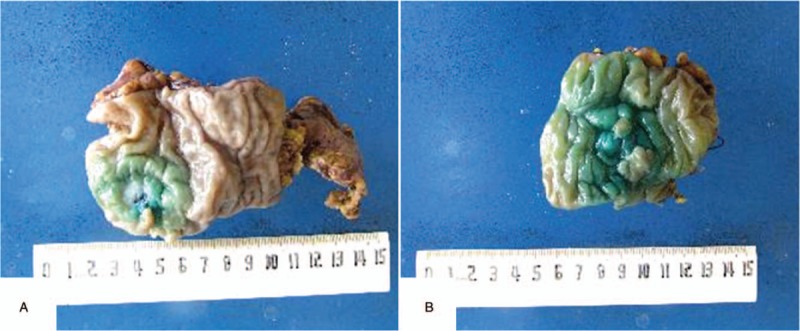
The tissue specimens of excised from colorectal. The distances from the anus were (A) 60 cm and (B) 10 cm, respectively.

**Figure 7:**
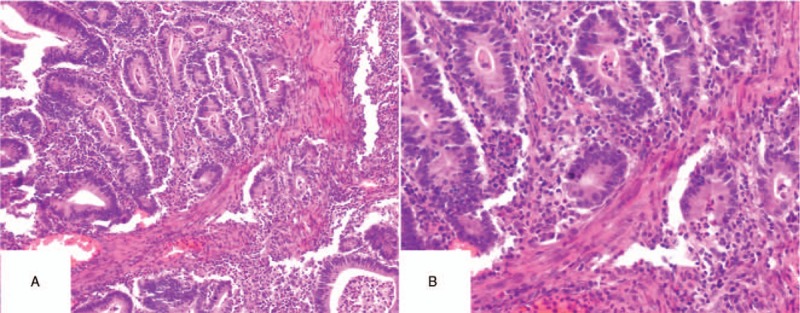
The HE staining of colon cancer tissue. the microscope magnifying × 40, (B) the microscope magnifying × 100.

**Figure 8:**
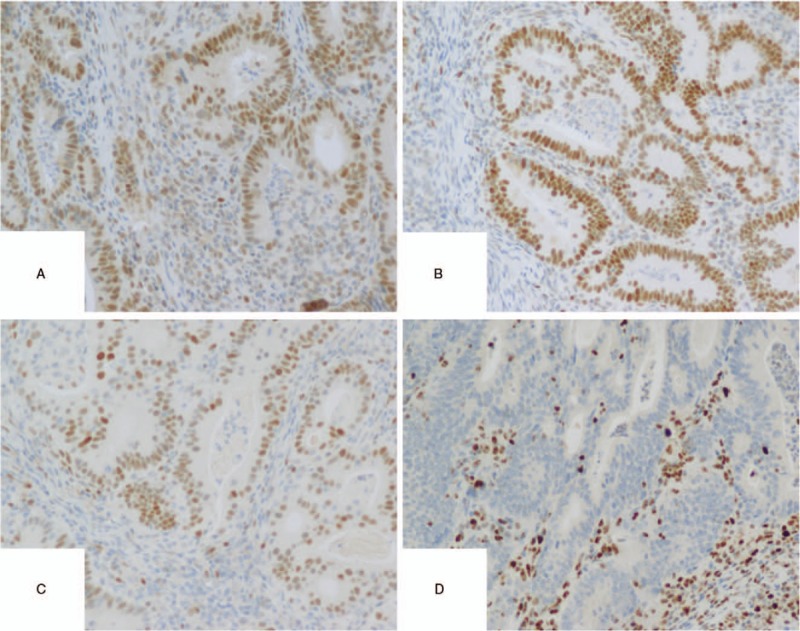
The IHC of colon. (A) MLH1 results showed positive nuclei, (B) MLH2 results showed positive nuclei, (C) MSH6 results showed positive nuclei, (D) PMS2 results showed negative nuclei.

During the 5-year follow-up, no recurrence of gynecological tumors was reported. The results of gene detection (Sanger Deoxygenation Chain Termination Method) were as follows (Table 1): a frame shift mutation c.1577 delA (p.Asp526 Alafs^∗^69) was detected in PMS2 gene of the subject (Fig. 4A-B). The mutation resulted in premature termination of the coding protein at the site of 594, thus leading to the truncation of the polypeptide chain, while the normal gene could encode 862 amino acids (Table 2). Colonoscopy revealed 2 polyps in the transverse colon, about 1.0 cm and 2.5 cm in size, respectively (Fig. 5A–D). Laparoscopic partial enterectomy was performed under general anesthesia for treating colon cancer 5 years later after the surgery treatment for EC. Pathological diagnosis (Figs. 6–7): Highly differentiated adenocarcinoma of the transverse colon, PTNM stage: T1N0Mx (Note: PMS2 IHC staining is negative). IHC staining results (Fig. 8): BRAF V600E mutation specific antibody (VE1) (Ventana IHC enhanced amplification kit) (−), PMS2 (−), EGFR (+), CDX2 (+), Ki67 (positive rate 90%), P53 (scattered +), MLH1 (+), MSH6 (+), MSH2 (+), CD31 (−), D2–40 (−).

**Table 1:**
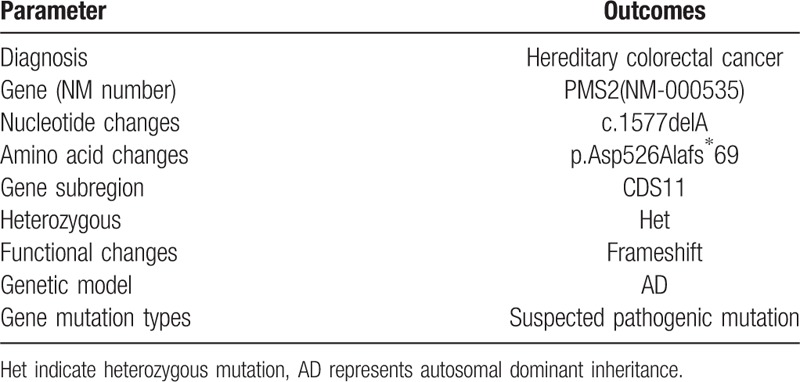
Gene detection of hereditary colorectal cancer.

**Table 2:**
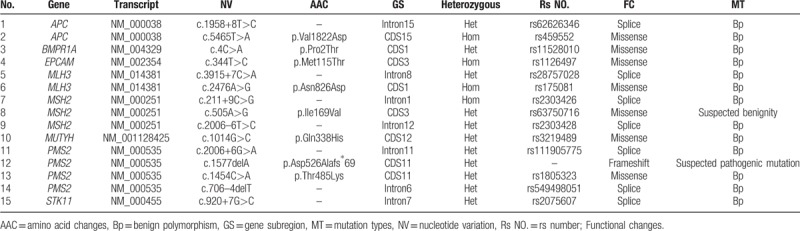
Variation information of exon region and its adjacent (+10 bp) intron region in hereditary colorectal cancer.

### 3.2 Family history

**Figure 9:**
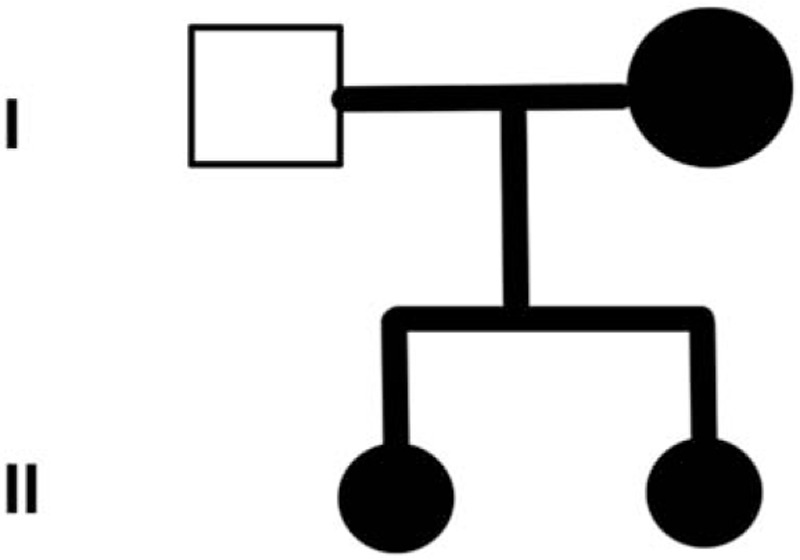
Pedigree structure of this Chinese family and validation of the PMS2 (NM_000535) mutation by Sanger sequencing. The family members that suffered from tumors are indicated with shading. Squares and circles denote males and females, respectively. Roman numerals indicate generations.

**Figure 10:**
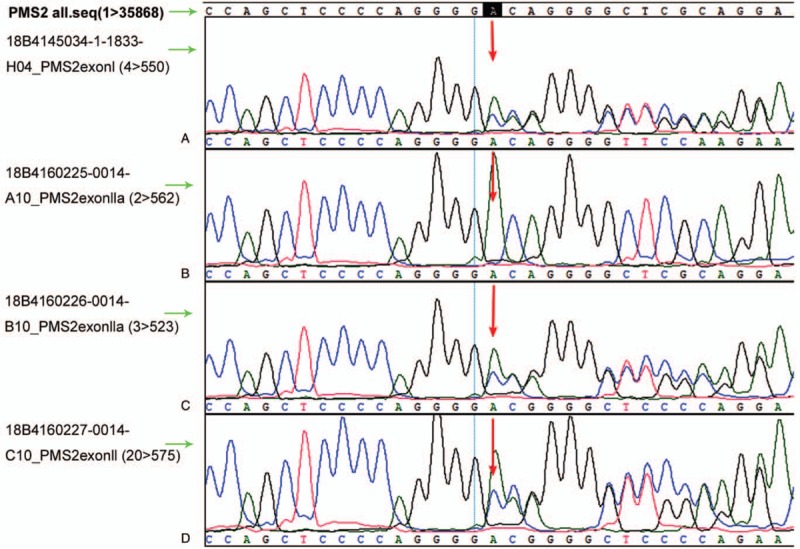
The results of family gene detection were analyzed by Sanger method. (A) Heterozygous mutations were observed in the patient, (B) Genetic tests on the patient's father showed normal results, (C) Heterozygous mutations were observed in the patient's mother, (D) Heterozygous mutations were observed in the patient's sister. The red arrows show the heterozygous mutation c.1577delA (p.Asp526Alafs^∗^69).

The patient's sister was diagnosed with EC at the age of 31, her mother diagnosed with rectal cancer at the age of 42, and his grandfather died of liver cancer. In order to confirm the mutation of the family, genetic counseling and analysis were carried out among the family members of the patient using Sanger's double deoxygenation method. The results showed that the sister and the mother carried the mutation (Figs. 9–10). Some family members refused the request, so no genetic analysis was performed in them.

## 4 Discussion

Colorectal cancer is a common malignant gastrointestinal cancer.^[11]^ Hereditary non-polyposis colorectal cancer (HNPCC) and familial polyposis are common susceptibility syndromes of hereditary colorectal cancer. HNPCC syndrome, also known as LS, is mainly caused by mutations in DNA MMR (MLH1, MSH2, MSH6 and PMS2). The risk of colorectal cancer, EC and ovarian cancer is as high as 80%, 20% to 60% and 6% to 13% respectively. MLH 1 and MSH2 mutations account for 90% of LS, MSH6 mutations for 10% and PMS2 mutations for 6%.^[6]^ In previous studies, there are many reports about MLH1 and MSH2 mutations, occasionally MSH6 mutations can be reported in LS, while PMS2 mutations are very rare. In this paper, a case of germline mutation c.1577delA (p.Asp526Alafs^∗^69) in LS is introduced.

In the past, the mutation of PMS2 prevalence was underestimated, indicating that PMS2 is not a critical susceptibility gene of LS. The importance of LS has been questioned by an unclear risk for extra-colonic cancers,^[12]^ the atypically low cancer penetrance in PMS2 families,^[13]^ as well as inconsistencies across studies due to variable cohort sizes and carrier ascertainment.^[14]^ It is because of these factors that the surveillance methods of PMS2 are still in controversy.^[12,15]^

PMS2 is central in the postreplicative human DNA mismatch repair (MMR) mechanism.^[16]^ This gene mutation can lead to the loss of mismatch repair function, and ultimately affect the proliferation and regulation of normal cells, thus resulting in cancer susceptibility. A frame shift mutation c.1577 delA (p.Asp526 Alafs^∗^69) was detected in the PMS2 gene of the patient. This mutation resulted in the early termination of 594 sites of the protein encoded by the PMS2 gene, causing the truncation of its polypeptide chain, while the normal gene could encode 862 amino acids.

LS can be divided into type I and type II according to the location of tumors. Type I is an intestinal neoplasm mainly composed of colorectal cancer, while type II is a colorectal cancer complicated with parenteral neoplasms, including EC, epithelial ovarian cancer, breast cancer, bladder cancer, renal cancer and gastric cancer. This patient suffered from colon cancer and EC successively, belonging to type II LS.

LS-related EC has the following characteristics:

(1) The age of onset is about 46 to 54 years old.
(2) Pathological types are diverse and poorly differentiated.
(3) Most of the lesions are located in the lower segment of the uterine body.

Studies have shown that in patients with LS-related EC, the risk of developing the second type of cancer is about 25% in 10 years and increases to 50% in 15 years.^[17]^ The median time for LS patients with ovarian cancer as the first disease to develop another type of cancer is 5.5 years. The median time for LS patients with ovarian cancer as the first disease to develop another type of cancer is 5.5 years. In gynecological diseases, patients with EC and ovarian cancer are highly suspected with LS if they have the above characteristics of LS-related gynecological tumors and have family or personal history of LS-related tumors. In this case, the onset age of ES was 26 years old. The onset age of LS was 31 years old. Because the patient knew very well that she had a family history of LS, she was diagnosed in an early stage of LS due to periodical gastrointestinal endoscopy, which is very helpful to the prognosis.

For patients with highly suspected LS-related gynecological tumors, the personal history, family history, menstrual status and childbearing history, family cancer history and the history of hereditary diseases should be collected in details and comprehensively. In practices, protein staining is easier and more economical than DNA analysis. IHC was used to detect MR protein and MSI in tissue samples for extensive screening. The consistency of MSI gene mutation detection and IHC detection are very high.^[18,19]^ The patient's surgical specimens were examined for MMR protein IHC, if the results show that there are protein deletions of MSH2, MSH6 and PMS2, it is necessary to further detect the corresponding protein deletion genes to determine whether there is a MMR mutation. If the MLH1 protein is deleted in surgical specimens, it is necessary to continue the detection of B-raf (B-Raf proto-oncogene, BRAF) oncogene and promoter methylation of MLH1 gene in tumor tissues, this is because about 75% of patients with MLH1 protein deletion are not LS patients and the loss of MLH1 protein in surgical specimens is usually caused by promoter methylation of MLH1 gene. In addition, patients with LS colorectal cancer rarely carry BRAF gene, while those without LS colorectal cancer have a mutation rate of 68%.^[20]^ The specificity of MSI in patients with MMR mutations is 90%,^[18]^ The sensitivity of detection is 80% to 91% in patients with MLH1 or MSH2 gene mutation and 55% to 77% in patients with MSH6 or PMS2 gene mutation. The sensitivity and specificity of IHC in patients with MR mutations are 83% and 89%.^[18]^ When the results of IHC and MSI tests indicate highly suspicious LS, it is recommended that those patients should undergo genetic testing. In this case, gene detection of several relatives of the patient revealed that the mutation site was located in PSM2, which was a great help to the follow-up treatment, and also warned relatives to take active preventive measures.

At present, there are few studies about LS-EC. It is hoped that this report will draw enough attention of obstetricians and gynecologists to LS-EC and large-scale, multi-center studies can be carried out to get more information about the incidence, clinicopathological characteristics, genetic changes, prognostic relevance and predictive values of the treatment of LS-EC among Chinese population.

## Author contributions

**Conceptualization:** Tong Yu.

**Methodology:** Dong-Wei Huang, Yan Jia.

**Project administration:** Dong-Wei Huang.

**Validation:** Dong-Wei Huang.

**Writing – original draft:** Xi-Wen Zhang, Tong Yu, Yan Jia.

**Writing – review & editing:** Man-Hua Cui, Yan Jia.
